# Highlight: How Mixing and Matching Alleles Shaped Human Ancestry

**DOI:** 10.1093/gbe/evad077

**Published:** 2023-05-18

**Authors:** Casey McGrath

The course of human history has been marked by complex patterns of migration, isolation, and admixture, the latter a term that refers to gene flow between individuals from different populations. Admixture results in a blending of genetic lineages, leading to increased genetic diversity within populations. In addition to admixture among modern human populations, ancient humans reproduced with other hominin groups, such as Neanderthals and Denisovans. This resulted in fragments of DNA from these ancient lineages being passed down to modern humans in a process known as introgression. Two recent studies published in *Genome Biology and Evolution* examine patterns of admixture in two different regions of the world—Africa and the Americas—revealing how this process has shaped the genomes of modern humans.

Africa is the birthplace of humanity, where our species originated and diversified. Because of this, Africa contains the highest levels of genetic diversity and population structure among humans, with non-African populations largely representing a subset of the genetic variation present on the African continent. Genomes of Africans contain mixtures of multiple ancestries, each of which has experienced different evolutionary histories. In the article “Evolutionary genetics and admixture in African populations,” researchers from two institutes—Georgia Institute of Technology and Mediclinic Precise Southern Africa—reviewed how multiple demographic events have shaped African genomes over time (Pfennig et al. 2023). According to Joseph Lachance, one of the review's authors, “What stands out is the sheer complexity of human demographic history, especially in Africa. There are many examples of population divergence followed by secondary contact, the legacy of which is written in our genomes.”

For example, ancient introgression from archaic “ghost” populations of hominins that are no longer extant contributed ∼4–6% of the ancestry of present-day Khoe-San, Mbuti, and western African populations. More recent demographic events that have occurred over the last 10,000 years have similarly resulted in admixture among modern humans, including gene flow among different click-speaking Khoe-San populations, the spread of pastoralism from eastern to southern Africa, and migrations of Bantu speakers across the continent.

Importantly, biomedical studies often fail to capture this diversity, resulting in implications for the health and disease of those with African ancestry. A better understanding of genetic architecture can help predict disease risk in a population or even inform clinical decision-making for individual patients. Such information is critical for equitable biomedical research, leading the study's authors to call for more ethically conducted studies of genetic variation in Africa. “A critical point right now is the relative lack of African genetic data,” says Lachance. “Most genomic studies have focused on Eurasian populations, and this limitation can exacerbate existing health inequities.”

One avenue for better understanding, the genetic architecture of African genomes is the study of ancient DNA: “Going forward, analysis of ancient DNA is expected to become much more common. Future studies are also likely to focus on fine-scale population structure in Africa. However, logistical and financial obstacles persist. There is a clear need for funding mechanisms that build research capacity in Africa.”

A second article recently published in *Genome Biology and Evolution*, titled “The impact of modern admixture on archaic human ancestry in human populations,” focuses on admixture in the Americas (Witt et al. 2023), which were colonized by modern humans relatively recently. The first people to enter the continent were Indigenous Americans who migrated from Siberia. Subsequent migration of Europeans and Africans due to European colonization and the Transatlantic slave trade resulted in admixed populations that combine ancestries from different continents.

In the study, researchers from Brown University, the Universidad Nacional Autónoma de México, and the University of California-Merced analyzed how the resulting gene flow between modern humans redistributed archaic ancestry in admixed genomes. They used data from the 1,000 Genomes Project that were acquired from several admixed populations, including Colombians from Medellin, individuals with Mexican Ancestry from Los Angeles, Peruvians from Lima, and Puerto Ricans from Puerto Rico. These genomes were compared with the high-coverage genomes of Neanderthals and Denisovans, ancient hominins that diverged from modern humans about 500,000 years ago and mated with humans in Eurasia before going extinct about 40,000 years ago.

According to one of the study's authors, Kelsey Witt from Brown University, these admixed populations are relatively understudied compared to more homogeneous populations. “It is common in studies like this for admixed populations to be excluded because the multiple ancestry sources can make those questions harder to answer. For this work, we wanted to focus on admixed populations to determine what we could learn from them, and whether admixed populations could provide information about all of the ancestry sources that contributed to them.”

The study found that the amount of introgression from Neanderthals and Denisovans was proportional to the amount of Indigenous American or European ancestry in each population. Although European and Indigenous American tracts in these admixed genomes have approximately equal proportions of Neanderthal variants, Denisovan variants are found primarily in Indigenous American tracts. This reflects the shared ancestry between Indigenous Americans and Asian populations, which also have higher levels of Denisovan introgression.

Moreover, by searching for archaic alleles present at high frequency in admixed American populations but low frequency in East Asian populations, the study's authors identified several genes as candidates for adaptive introgression. These genes were related to multiple pathways including immunity, metabolism, and brain development. Such findings have potential implications for the health of individuals in these admixed populations. “We’ve seen many examples of genetic mismatch in the literature,” says Witt, “where some variants were adaptive at some point in the past, but in the present environment, they have a negative impact on health. In addition, in admixed populations, genetic variants that are unique to separate populations may now interact in unexpected (sometimes negative) ways when they are present in the same individual. Our work suggests that some archaic variants are specific to some ancestry sources and not others.”

Like Lachance, Witt knows that additional research is needed to continue to untangle the effects of admixture on modern humans. “In a lot of ways, admixed populations in the Americas are straightforward to study because we have a good idea of the timing and number of gene flow events,” notes Witt. “I’d like to apply this work to other admixed populations, where we may not know when admixture occurred or which populations contributed to it, or in cases where the contributing populations are more closely related. I think that the answers in those cases may not be as clear-cut, but they may contribute to a better understanding of those recent admixture events.”

These studies show admixture has played a significant role in shaping human evolution, both in Africa and in the Americas. Admixture not only reshuffles the genetic variation within and between populations, but also introduces new sources of variation that may have adaptive potential. By comparing the genomes of admixed populations with those of their ancestral groups and with those of archaic humans, these studies reveal how the mixing and matching of alleles has shaped the evolution of our species.
